# Advancing health research practices among forcibly displaced populations: A multidisciplinary stakeholder workshop

**DOI:** 10.1186/s12961-026-01446-9

**Published:** 2026-01-27

**Authors:** Ameera S. Abu-Khalil, Rashmina J. Sayeeda, Beenish Shaikh, Alaaddin Salih, Sylvia Omulo, Esther Amulen, Justine N. Bukenya, Dathan M. Byonanebye, Neila Gross, Most Jesmin Ara Joly, Edward K. Kirumira, David Lubogo, Akhterul Kabir Munna, Olivia Esther Nakisita, Emmanuel Ntale, Michael T. Wagaba, Carly Ching, Maia C. Tarnas, Christopher Orach, Muhammad H. Zaman

**Affiliations:** 1 Department of Biomedical Engineering, Boston University, 44 Cummington Mall, Boston, MA 02215 USA; 2 Center On Forced Displacement, Boston University, Boston, MA USA; 3 Wellcome Trust, London, UK; 4 National Academy of Health Sciences, Khartoum, Sudan; 5 Center for Human Rights and Humanitarian Studies, Watson Institute for International and Public Affairs, Brown University, Providence, RI USA; 6 Paul G. Allen School for Global Health, Washington State University, Pullman, WA USA; 7 Makerere University School of Public Health, Kampala, Uganda; 8 Department of Materials Science and Engineering, Boston University, Boston, MA USA; 9 Médecins Sans Frontières (MSF)-OCA, Cox’s Bazar, Bangladesh; 10 Stellenbosch Institute for Advanced Study, Stellenbosch, South Africa; 11 Climate and Environment Unit, Uganda Red Cross Society, Kampala, Uganda; 12 Department of Population Health and Disease Prevention, University of California, Irvine, Irvine, CA USA

**Keywords:** Displaced populations, Refugees, Infectious disease, Research ethics, Participatory research methods

## Abstract

Health research among forcibly displaced populations presents distinct and multifaceted challenges, including limited healthcare access, heightened exposure to environmental hazards and insufficient research infrastructure. In December 2024, a multidisciplinary workshop convened in Kampala, Uganda, bringing together researchers, healthcare professionals, representatives from humanitarian non-governmental organizations and a global health funding body. The workshop aimed to identify key barriers and co-develop actionable strategies for conducting ethical and equitable research in contexts of displacement, with a specific focus on infectious disease. Across the 20 workshop participants, several critical challenges were identified: the misalignment between global health funding priorities and those of low- and middle-income countries; structural and methodological barriers in research design, such as restricted data access and the perpetuation of epistemic biases; and the ethical complexities of working with vulnerable and highly mobile populations. Discussants emphasized the essential role of sustained community engagement, transparent and bidirectional communication and targeted capacity-building as prerequisites for addressing these barriers. Proposed solutions highlighted the importance of long-term, sustainable research models supported by contextually adaptive methodologies, participatory approaches that centre community co-creation and the strengthening of regional research networks to improve access to funding and resources. These findings provide a basis for developing future frameworks aimed at improving health outcomes among forcibly displaced populations, and underscoring the need for a paradigmatic shift towards more inclusive, context-sensitive health research.

## Introduction

The health of refugees, displaced persons and migrants is an emerging global health priority, particularly in the context of infectious diseases [1–3]. Displaced individuals face a higher risk of contracting infectious diseases due to barriers in accessing healthcare, increased exposure to environmental hazards, inadequate housing and insufficient water, sanitation and hygiene (WASH) infrastructure [2]. Infectious diseases represent a critical and well-documented entry point for examining research practices in forced displacement settings, given their sensitivity to infrastructure, mobility and ethical constraints. The unique challenges associated with forced displacement necessitate a specialized research approach. To ensure equitable and high-quality care for these populations, research must adopt a synergistic, multidisciplinary and community-centred approach that addresses dynamic and pressing issues while recognizing the limitations of current practices. A critical evaluation of existing methodologies and the development of ethical, robust research frameworks are essential for enhancing research design, execution and dissemination. Accordingly, researchers from Boston University’s Department of Biomedical Engineering and Center on Forced Displacement in collaboration with the Wellcome Trust and Makerere University’s School of Public Health organized a multidisciplinary stakeholder workshop titled “Advancing Ethical Infectious Disease Research Practices among Forcibly Displaced Populations.”

Previously, the team at Boston University conducted a systematic review to evaluate the state of primary research on the prevention, control and management of infectious diseases within refugee camps and urban informal settlements in the top 10 refugee-hosting low- and middle-income countries [4]. The review identified several challenges associated with conducting research in settings populated by displaced persons, including high population mobility, limited external validity, interruptions, spillover effects and low recruitment rates. Notably, it highlighted a lack of comprehensive guidelines and frameworks for facilitating research and evaluating study quality in these settings [4]. To further explore existing tools, the team synthesized and compared current research frameworks to assess their utility, identify gaps and determine whether existing guidelines adequately address the challenges identified in the systematic review. The analysis revealed that the frameworks did not sufficiently account for the nuances between various types of displaced populations in crucial aspects of ethics and research. Moreover, these frameworks were not widely used for research in forced displacement contexts. Since displaced populations such as refugees, stateless persons, internally displaced persons (IDPs), asylum seekers, those affected by acute emergencies or conflicts and those living in protracted crises face diverse challenges, they require distinct, adaptable approaches tailored to their specific circumstances and needs [5–7]. Thus, a holistic framework must consider the complexities and nuances of displacement to effectively meet these needs.

To gain deeper insights into the challenges faced by local researchers for future research framework development, a collaborative, in-person workshop was convened to identify the obstacles to implementing research on infectious diseases in complex settings. While the workshop was initially oriented around infectious disease research, participants consistently identified challenges and proposed solutions that applied to research more broadly across health and humanitarian contexts. As such, the themes discussed and the resulting synthesis extend beyond infectious-disease-specific research and traditional applications of research ethics. The objectives of the workshop were to (i) build a coalition of partners who are interested in enhancing research involving displaced persons; (ii) conduct a stakeholder assessment of research needs with a focus on infectious disease; (iii) develop a prioritized list of changes to improve research; and (iv) communicate the workshop discussions and action items to the broader research and practitioner community. Through interactive discussions, the workshop also facilitated discussions on potential solutions. Here, we present the outcomes of this workshop.

## Methods

On 17–18 December 2024, a collaborative workshop employing a participatory approach was held at Makerere University School of Public Health in Kampala, Uganda. The workshop brought together diverse stakeholder groups, including researchers, academics, healthcare practitioners, members of institutional research ethics committees and representatives from funding agencies, humanitarian organizations and non-governmental organizations (NGOs) to foster a collaborative and comprehensive discussion (Table 1).

**Table 1:** Workshop discussant information

Stakeholder group^*^	Affiliated institution^**^	Research locale^***^	Years of experience
Academic, *N* = 14	Africa, *N* = 10	Africa, *N* = 13	0–2 years, *N* = 2
Ethical review board, *N* = 2	Europe, *N* = 3	Asia, *N* = 6	2–5 years, *N* = 5
Funding, *N* = 2	North America, *N* = 6	Europe, *N* = 1	6–10 years, *N* = 6
Healthcare, *N* = 7		North America, *N* = 2	> 10 years, *N* = 5
NGO, *N* = 3			

^*^Workshop discussants may belong to multiple stakeholder groups

^**^Workshop discussants may hold positions at more than one institution

^***^Research may have been conducted in various locations

This workshop did not require approval from an ethics committee, as it did not involve medical research with human participants. Participation was voluntary, and the workshop adhered to the ethical principles outlined in the Declaration of Helsinki. All participating discussants provided verbal informed consent at the onset of the workshop for the transcription of discussions. Verbal consent was deemed appropriate given the open and interactive nature of the discussions. Workshop facilitators (A.S.A., R.J.S.) recorded consent and reaffirmed it throughout the review and approval process of the workshop summary prior to submission.

Prior to the in-person convening, discussants completed a brief pre-workshop questionnaire about their experiences and perspectives on conducting research in forced displacement and refugee camp settings. The questionnaire included open-ended questions addressing the discussants’ research experience in these contexts, perceived ethical and logistical challenges, opportunities for improved research design and implementation, research guidelines or frameworks currently in use and recommended pre-reading materials. To inform the workshop agenda and discussion prompts, responses were reviewed and analysed qualitatively by the facilitators prior to the workshop to identify recurring themes and shared concerns across participant’s experiences.

This workshop was one component of an ongoing body of work, including a systematic review titled “Infectious disease research in forcible displaced populations: A systematic review in low-and middle-income countries” and a manuscript titled “Mapping utility and applicability of research and ethics frameworks for displaced populations” [4, 8]. Both were presented at the workshop to ground the discussion in existing evidence and inform workshop dialogue where the discussants have lived experience conducting research with displaced populations.

The workshop consisted of both large-groups discussions and smaller breakout sessions. This combination of methods brought to the surface common challenges and diverse perspectives that informed the workshop findings. In the primary large-group discussion, all discussants actively engaged in brainstorming the common points of contention identified in the pre-workshop questionnaire. These issues included the following:

1. What are the most significant challenges faced when conducting infectious disease research in refugee camps and displaced populations?
2. What factors contribute to the existence of these challenges and needs? What operational, logistical and structural barriers impede progress?
3. What are the primary research needs/questions?

Following this, a large-group session focused on funding processes and the research interests of funding organizations was conducted. The guiding questions included:

1. What funding gaps are perceived by workshop discussants?
2. What trends are evident in funding calls and potential future opportunities?

Finally, the discussants broke out into smaller groups to collaboratively develop a prioritized list of structural design changes intended to enhance health research in complex settings, guided by the following discussion prompts:

1. What practical strategies can we implement to address these challenges and needs?
2. How can we conceptualize, operationalize and establish an ethical research framework?
3. Analyse the applicability of proposed solutions or frameworks in specific contexts, and identify potential points of failure.

The breakout sessions allowed for more focused, in-depth conversations, after which each group reported back to the full workshop. This combination of methods brought to the surface common challenges and diverse perspectives that informed the workshop findings.

## Results and discussion

Here, we present convergent themes that emerged during group discussion about research challenges and opportunities for structural design changes in complex settings. Table 2 summarizes the underlying drivers of research challenges in complex settings, such as the structural and contextual factors responsible for shaping how research is planned and conducted (for example, bureaucracy, community relations, data access). Table 3 maps those drivers onto problem areas and potential mitigation strategies. The narrative that follows first highlights the root drivers of research challenges (Table 2) and then discusses opportunities for structural design changes that address these challenges (Table 3), showing how each set of solutions addresses one or more upstream drivers.

**Table 2:** Key drivers of challenges in complex health research settings

Theme	Responses
Bureaucracy	Individual researchers face difficulties in obtaining grants or publishing findings. Negotiating with stakeholders becomes complex when resources are controlled by governmental or military entities. Limited engagement occurs in the pre-proposal process. Institutional review board (IRB) processes are often slow, multilayered and biased, frequently lacking expertise in specific topics. Context-specific obstacles and adaptations hinder standardization.
Community relations	Managing community expectations poses significant challenges. Intra-community social dynamics greatly influence research efforts. Support structures for research within institutions and communities remain underdeveloped. Communities may harbour distrust towards researchers or experience fatigue from repeated research initiatives.
Research challenges	Gaps in language and cultural competency impede effective communication. Researchers often juggle multiple roles, leading to fatigue. Training stable research teams within dynamic settings presents considerable challenges. Population segregation introduces biases in longitudinal studies.
Data access and collection	There is a lack of common, accessible databases for researchers and communities. Budgetary constraints, bureaucratic hurdles and motivational issues restrict access to district-specific information. Assessing the quality and accuracy of data proves difficult United Nations High Commissioner for Refugees (UNHCR)-associated health partners often restrict access to their databases, hindering information sharing. Political factors limit data access, resulting in publication tensions. Fixed data creates pressure for timely publication. Local publications are available but often do not meet international standards.

**Table 3:** Potential opportunities for problem mitigation in complex health research settings

Problem area	Potential opportunities for mitigation
Stakeholder engagement	Developing intentional capacity through stakeholder coordination at all levels Training researchers in effective communication to advocate for institutional support Establishing a community of practice to enhance collaboration, share resources and address common challenges among researchers and practitioners
Research design	Identifying the core problem by thoroughly navigating the existing research landscape, including grey literature Emphasizing ideals such as humility, empathy and trust throughout the research design process Defining roles for personnel with the technical expertise required to improve research design practices across diverse stakeholders
Research facilitation	Engaging external expertise to gain insights into community dynamics Setting realistic expectations Training research teams for sustained engagement in their respective areas
Research dissemination	Improving the adoption of research findings and the integration of these findings into stakeholder practices Enhancing the visibility of research among peers and the community Identifying or establishing institutional platforms for publishing research related to refugees

### Research challenges and opportunities for problem mitigation

What are the challenges to health research in complex settings?

### Research design

Upon beginning a research project, workshop discussants agreed that literature reviews are a foundational step for understanding the current state of a research field. However, in the context of complex settings, such as within informal settlements and refugee populations, conducting these reviews is particularly challenging. Workshop discussants identified accessing relevant, centralized data as a common challenge in the literature review process prior to research design. They noted that reports often lack relevance because they rely on outdated or limited data, contain biases and use standardized methods that do not reflect the unique realities of local communities. For example, workshop discussants found that, in some countries, public health sectors face challenges in providing region-specific information, such as distinguishing between refugees and host populations due to limitations in data collection methods, which fail to capture essential variables related to community health issues needed for background on the diseases being studied in vulnerable settings.

Discussants emphasized the challenges researchers face with gaining funding for research in complex settings once the project had been shaped through the literature review process. Notably, workshop discussants emphasized that researchers in complex settings often grapple with issues of credibility, as well-funded research typically focuses on more insular, established perspectives. Such drivers of siloed research agendas further perpetuate the cycle that overlooks the need for lived experience that informs research design and instead focus on a researcher’s prior work. According to the workshop discussants, few possess the technical or material capacity to invest in essential pre-proposal processes or pilot studies before applying to active grant calls. This inequity is compounded by administrative and bureaucratic hurdles, such as restrictive visa policies, institutional barriers and language requirements, which can slow or prevent access to funding opportunities [9–11]. In addition, short funding cycles rarely align with the realities of conducting longitudinal, community-engaged research in these environments, while power imbalances in international collaborations frequently position local researchers as subcontractors rather than leaders of research agendas. Thus, the review process introduces a bias by favouring methodological familiarity and track records over contextual expertise, reinforcing systematic disadvantages.

The final step before implementing research design is ethical review approval. Ethical review committees are crucial for safeguarding study participant safety and welfare in vulnerable populations [12]. However, they are often multilayered and heavily bureaucratized, frequently lacking specialized expertise [13]. In complex settings, workshop discussants emphasized the juxtaposition between the need for an expedited review process with the time it takes just to get an appointment with review board specialists. This poses a significant challenge for urgent studies, such as those related to infectious disease outbreaks.

### Research facilitation

Facilitating studies with vulnerable populations in complex settings presents a range of challenges that existing frameworks inadequately address. Workshop discussants noted that one of the most important aspects of research facilitation is communication between the researchers and study participants. Workshop discussants emphasized that, of the many intricacies of communication, language barriers can be particularly problematic. This is especially the case in refugee communities with diverse nationalities, such as in Uganda, where there are refugees from at least 34 countries [14]. In addition to the verbal language barriers, most vulnerable communities lack written language skills. Unclear intra-community communication dynamics hinder health networks, though community health workers can sometimes act as intermediaries. These structures remain underdeveloped and lack support in many low-resource settings, where workshop members expressed that researchers must balance additional roles as community workers, anthropologists, policy advocates and others, leading to fatigue.

In addition to communication-related challenges, researchers at the workshop described past difficulties managing community expectations, particularly when communities expect direct, immediate benefits from research findings. This issue echoes broader concerns in global health literature about the ethical tensions between research goals and community needs [15]. Consequently, communities often lack trust in researchers, particularly when research fatigue arises from numerous studies having been conducted, with little to no visible change since the research commenced [16]. On the basis of their direct experiences, many workshop discussants communicated that communities expect honesty from researchers, especially when their participation in studies is solicited; thus, over-promising translational interventions can erode trust. In some instances, communities may tailor their responses to align with researchers’ hypotheses, anticipating material benefits in return [17]. In economically disadvantaged communities, the expectation for incentives, particularly financial ones, has not been clearly addressed, creating a dual deterrent for both refugees and researchers, especially when funding is limited, as is often the case in refugee settings.

### Research dissemination

In complex settings, such as those affected by political instability or civil unrest, workshop discussants highlighted that certain data may need to remain unpublished or confidential. This can be a measure to avoid stigmatizing vulnerable populations or assigning blame to governing bodies for failing to protect them. This, in turn, can create tension among various stakeholders such as researchers, governing bodies and the vulnerable population in need of assessment and intervention. Moreover, discussants reflected that the quality and reliability of available data are often difficult to determine, as the absence of centralized databases, limited publication of findings and lack of coordinated discourse within the research community create a fragmented environment with few mechanisms for validation with no clear mechanisms for assessing or validating its reliability. Additionally, the limited dissemination of research findings to communities makes it increasingly difficult to engage relevant stakeholders in the study outcomes and solicit feedback. Workshop discussants also noted that local publications, while more accessible, frequently do not meet international standards for broader dissemination. This raises a critical question: Where, then, should this type of research be published?

What are the opportunities for structural design changes that will improve health research in complex settings?

### Stakeholder engagement

Stakeholder engagement was identified as essential to shaping research design, rather than as an activity limited to later stages of the research process. Workshop discussants emphasized that engaging key stakeholders, including affected communities, local researchers, implementing partners, governments and ethics bodies, during problem formulation and study design can mitigate many of the structural challenges identified in Table 2, such as bureaucratic delays, restricted data access and community mistrust. Early engagement enables researchers to align study objectives with community priorities, anticipate ethical and operational constraints and improve feasibility within existing institutional structures. To achieve intentional stakeholder engagement, discussants emphasized that researchers must first understand bureaucratic processes and the priorities of key stakeholders, including governments, research institutions and the study population. Accordingly, discussants highlighted the need for training in effective communication to secure institutional support and foster collaboration. Sustained engagement with stakeholders can improve the clarity, efficiency and long-term impact of research, even when findings are not ultimately published.

While these principles apply broadly, discussants noted that the specific challenges researchers face often vary by regional context and the nature of the crisis. Contextual challenges differ by region and research type, particularly between acute emergencies and protracted crises [18]. To address these challenges, discussants proposed establishing communities of practice for researchers and practitioners to exchange solutions, minimize duplicated efforts and alleviate research fatigue. Strategic collaborations that would address these contextual challenges are essential for researchers in the Global South to gain access to funding and resources. Discussants also advocated for a multidisciplinary approach to address existing inequities, including the creation of regional funding opportunities and the promotion of ongoing collaboration, while engaging disciplines such as sociology and medical anthropology to better understand community dynamics and support more ethically grounded research practices.

### Research design

Discussants largely concurred that research should prioritize long-term impact over transient project engagements. While identifying the appropriate research question is an essential first step, a thorough examination of existing research requires access to both formal evidence and practice-based knowledge. Literature reviews establish context, guide methodological choices, foster innovation and prevent the duplication of efforts [19]. While traditional literature reviews help draw a perimeter of existing scholarly work, they are insufficient for capturing locally generated knowledge that shapes ongoing interventions but rarely appears in peer-reviewed outlets. As a structural opportunity, discussants highlighted the value of establishing aforementioned communities of practice or local research consortia that bring together researchers, policy-makers and community members. These platforms can function as living repositories of experiential knowledge, documenting lessons learned, failed approaches and contextual constraints that are not easily captured through conventional academic publication. Unlike grey literature collected retrospectively, such collaborative infrastructures enable real-time knowledge exchange and reduce redundancy by making ongoing and unpublished work visible across actors operating in similar settings. When communities are engaged as co-designers of research questions and study designs, these collective structures help ground research priorities in lived experience and implementation realities. This approach shifts the burden of “scanning the field” away from individual research teams and towards shared, relational systems of knowledge production, strengthening the relevance and long-term impact of such research.

In response to the complexity and unpredictability of working with mobile or vulnerable populations, discussants emphasized the need for research designs that are both scientifically rigorous and operationally adaptive. For instance, designs should include risk assessment frameworks that anticipate disruption or logistical breakdowns, enabling timely adjustments without compromising data integrity. Responsive methodologies with real-time monitoring support adjustments in sample accessibility, remote data collection and staged ethical approvals. Modular or gated protocols where each phase yields stand-alone insights can ensure data utility even if a study is halted early. Moreover, partial completion benchmarks can help funders and ethics bodies recognize the value of incomplete datasets, reducing both participant burden and resource waste. Early phases, for example, may focus on baseline descriptive data with independent policy relevance. Similarly, budgets should account for buffer time, rerouted plans and additional human resources (for example, for follow-up or re-consent), reflecting the relational and logistical demands of complex settings.

### Research facilitation

In complex, community-based settings, research facilitation encompasses the processes, structures and roles that enhance both the effectiveness and ethical integrity of research. A key responsibility identified was the accountability of researchers to vulnerable communities, which is crucial for fostering impactful research. To support this accountability, workshop discussants emphasized the importance of co-created training programs, especially given that many of the challenges outlined above in project design can hinder research efforts. Additionally, principal investigators and research advisors must proactively allocate time and resources to support these particular research team roles, ensuring effective communication and collaboration between researchers and the community.

In addition to training programs, discussants agreed that, where possible, protocols should align with existing local systems such as health, education or social services to support continuity and sustainability. Community health workers (CHWs), as trusted and culturally fluent members of the community, can help minimize disruption and facilitate effective communication, unlike institutional researchers who may be viewed as outsiders [20]. While not necessarily a novel approach, the workshop discussants highlighted the value of involving CHWs in training and delegating study-related tasks to them or similar community-based roles. This can strengthen community relationships, reduce researcher burden and improve the uptake of research findings.

Complementing these structural approaches, workshop discussants emphasized that researchers themselves must actively guard against research fatigue by approaching their work with humility and empathy, setting realistic expectations and avoiding over-promising outcomes. Building trust with community participants begins at the first stages and extends throughout the entire research process. Discussants also agreed that peer accountability can be strengthened by clearly defining the roles of relevant experts, ensuring they contribute meaningfully to research design, ethical reviews and funding decisions. Central to this process is a commitment to ethical frameworks that guide research principles with vulnerable people, which not only fosters trust but also ensures the accuracy of findings and reduces the risk of harm to communities involved.

### Research dissemination

Ensuring the visibility of research is critical, allowing communities to access and comprehend findings, especially regarding language, future study directions and policy implications. Disseminating these findings to key stakeholders, including government bodies and affected communities, fosters sustained support and trust among all parties. In this vein, structured data archiving, metadata documentation and clear data-sharing protocols should be standard. These measures ensure transparency, support reproducibility and allow future research to build on partial results, making the research effort cumulative, even if disrupted in crisis-affected settings. To address the absence of platforms for research in these contexts, some workshop discussants suggested creating additional journals focused on low- and middle-income countries (LMICs) leading refugee research. Others proposed establishing and maintaining working groups tailored to specific conflicts, regions or issues.

### Addressing funding gaps in global health research

Current global health funding structures often present significant challenges for researchers, particularly those in Global South (or low- and middle-income) countries [21, 22]. Some workshop discussants noted that funding bodies often prioritize adherence grant application specifications over more contextually relevant research designs, making it difficult to secure support that would better address local health needs. For example, a researcher in Senegal might be restricted to malaria-focused pharmaceutical research due to funding restrictions, even though tuberculosis is also prevalent in the region. This narrow, issue-specific approach not only silos health issues but also limits the potential for addressing comorbidities and broader health challenges.

The imbalance of power in global health research funding continues to shape which resources are allocated, how knowledge is generated and which priorities are addressed. Most funding originates from high-income countries (HICs), often misaligned with the actual burden of disease in low- and middle-income countries (LMICs) [21]. Influential donors, including governments from the USA and Europe, alongside major philanthropic organizations such as the Bill & Melinda Gates Foundation, the World Bank and the Global Fund, play a dominant role in setting the global health research agenda [23, 24]. This concentration of influence limits the ability of researchers from LMICs to lead initiatives that reflect their regions’ most pressing health challenges and needs.

The funding landscape is disproportionately controlled by high-income countries, creating significant barriers for researchers in the Global South, who often face bureaucratic, political and credibility obstacles when competing for grants [25]. Drawing on their first-hand experience, several workshop discussants noted that, even when their research design is robust, a lack of strategic alliances with Global North funding bodies can prevent them from accessing prominent funding opportunities. Moreover, drawing on their collective expertise, workshop discussants synthesized that the short-term, immediate crisis-driven nature of many funding streams further undermines sustainable solutions, particularly for forcibly displaced populations [26, 27]. Discussants reflected that funding rarely supports essential pre-study activities such as community engagement and trust-building, which are key to avoiding extractive research practices.

Table 4 outlines key challenges within the current funding landscape, alongside potential solutions for funders to adopt more equitable, effective approaches to global health research funding. Workshop discussants agreed that, by implementing long-term investments, prioritizing community engagement and fostering equitable partnerships, funders can play a pivotal role in transforming the global health research ecosystem to better serve those most affected by health inequities.

**Table 4:** Key challenges researchers face regarding funding and potential solutions funders can adopt

Key challenge	Explanation	Potential solutions
Restricted research focus due to funding priorities	Researchers often have to align with donor specifications, rather than addressing the most pressing health concerns in their regions. For example, a researcher in Senegal may be forced to focus on malaria even when tuberculosis is also a major issue.	Funders should prioritize flexible, needs-based funding that allows researchers to address local health priorities, rather than pre-defined donor agendas.
The Global North’s domination in funding and decision-making	Large funding bodies in high-income countries control most research funds, limiting opportunities for researchers in the Global South. Strategic alliances with Northern researchers are often necessary to secure grants.	Increase direct funding to researchers and institutions in the Global South. Foster equitable collaborations that prioritize Southern leadership and agenda-setting.
Short-term, crisis-driven funding	Many funding opportunities are tied to immediate crises (for example, coronavirus disease 2019 [COVID-19]), neglecting long-term structural investments.	Establish long-term, sustainable funding models that build research capacity and infrastructure over time.
Extractive research models	Communities are often studied, rather than engaged, leading to a lack of trust, fatigue and minimal long-term impact.	Allocate a portion of funding (10–15%) specifically for community engagement to ensure co-created research with local populations.
Barriers in conflict zones and politically sensitive areas	Research in conflict-affected areas (for example, Sudan, Yemen) is hindered by security concerns and a lack of established funding relationships.	Funders should develop risk-sensitive funding mechanisms and partnerships with local organizations to facilitate research in fragile settings.
Declining global health funding and shifting priorities	Donor fatigue and new crises (for example, the war in Ukraine) have diverted funds away from ongoing global health challenges, disproportionately affecting forcibly displaced populations.	Advocate for sustained, multi-sectoral investments in global health, ensuring that funds are not entirely reallocated with each emerging crisis.

## Limitations of this workshop

This work is based on discussions held during a single workshop, which naturally introduces limitations. The perspectives represented reflected the backgrounds and expertise of the participating researchers, practitioners and funding entities. While this diversity was valuable, the group composition may have carried inherent biases linked to disciplinary training, institutional affiliations and positionalities in the global research ecosystem. Importantly, the workshop did not include direct representation from patient or research subject groups, meaning that the voices of forcibly displaced individuals themselves were not directly present in shaping the dialogue. As such, the themes and recommendations reflect the lens of those who design, fund and implement research, rather than the lived experiences of those most affected. Future work might actively integrate displaced persons and this work into both the discussion and design of research approaches to ensure that proposed ethical frameworks are co-created and grounded in study participant realities.

## Conclusion

While the majority of workshop discussants share a background and/or research interests in infectious diseases, the workshop underscored the multifaceted challenges associated with conducting health research in refugee camps and among displaced populations. Discussants emphasized the necessity for more equitable access to research funding streams and opportunities, particularly for research and academic institutions based in the Global South. The diverse insights and perspectives shared by discussants highlight the importance of fostering open dialogue among various stakeholder groups, including researchers, practitioners, funding bodies and representatives from NGOs. Future directions should focus on establishing and supporting networks that connect interdisciplinary, multi-divisional, cross-regional and cross-continental research initiatives. New frameworks should encompass research coalitions, co-creation spaces and platforms for funding streams.

## Paper context

In regard to our main findings, informed by diverse stakeholders with broad expertise, the workshop identified key barriers to ethical and equitable infectious disease research with forcibly displaced populations, including inaccessible and misaligned funding, biased research design and ethical complexities to participatory research methods.

In terms of added knowledge, the workshop highlighted the need for tailored research frameworks that address the unique challenges of displaced populations and the importance of community engagement, co-creation and multidisciplinary stakeholder collaboration in overcoming these barriers.

In regard to global health impact for policy and action, these findings provide actionable steps forward to enhancing research practices and funding access, which can inform global health policies aimed at improving health outcomes for forcibly displaced populations, particularly in low- and middle-income countries.

## Data Availability

The authors confirm that the data supporting the findings of this study are available within the article.
